# Hip preserving surgery with concentrated autologous bone marrow aspirate transplantation for the treatment of asymptomatic osteonecrosis of the femoral head: retrospective review of clinical and radiological outcomes at 6 years postoperatively

**DOI:** 10.1186/s12891-017-1652-8

**Published:** 2017-07-06

**Authors:** Yohei Tomaru, Tomokazu Yoshioka, Hisashi Sugaya, Katsuya Aoto, Hiroshi Wada, Hiroshi Akaogi, Masashi Yamazaki, Hajime Mishima

**Affiliations:** 1 Department of Orthopaedic Surgery, Faculty of Medicine, University of Tsukuba, 1-1-1 Tennodai, Tsukuba, Ibaraki, 305-8575 Japan; 2 Division of Regenerative Medicine for Musculoskeletal System, Faculty of Medicine, University of Tsukuba, 1-1-1 Tennodai, Tsukuba, Ibaraki, 305-8575 Japan

**Keywords:** Osteonecrosis of the femoral head, Femoral head collapse, Low-invasive surgery, Bone marrow aspirate, Autologous transplantation

## Abstract

**Background:**

We had previously established concentrated autologous bone marrow aspirate transplantation (CABMAT), a one-step, low-invasive, joint-preserving surgical technique for treating osteonecrosis of the femoral head (ONFH). The present study aimed to evaluate the effects of CABMAT as a hip preserving surgical approach, preventing femoral head collapse in asymptomatic ONFH.

**Methods:**

In total, 222 patients (341 hips) with ONFH were treated with CABMAT between April 2003 and March 2013. Based on magnetic resonance imaging, we determined that 119 of these patients had bilateral asymptomatic ONFH (238 hips), and 38 further patients had unilateral asymptomatic ONFH (38 hips). In this series, we retrospectively examined 31 hips in 31 patients with unilateral asymptomatic ONFH treated surgically between 2003 and 2012 and followed up for more than 2 years. Clinical and radiological evaluation were performed immediately before the procedure and at the final follow-up. The two-year follow-up rate among patients with unilateral ONFH was 82% (31/38). Therefore, the present study included 31 patients (19 males and 12 females), with a mean age and follow-up period of 40 and 5.8 years, respectively. Of the 31 asymptomatic hips, 5, 6, 10, and 10 had osteonecrosis of types A, B, C1, and C2, respectively. The diagnosis, classification, and staging of ONFH were based on the 2001 Japanese Orthopaedic Association (JOA) classification.

**Results:**

Secondary collapse of the femoral head was observed in 6/10 hips and 5/10 hips with osteonecrosis of types C1 and C2, respectively. Total hip arthroplasty was performed in 9.6% of patients (3/31 hips), at an average of 33 months after surgery. Clinical symptoms improved after surgery, and the secondary collapse rate at a mean of 5.8 years after CABMAT was lower than that reported in several previous studies on the natural course of asymptomatic ONFH.

**Conclusions:**

Early diagnosis of ONFH (i.e., before femoral head collapse) and early intervention with CABMAT could improve the clinical outcome of corticosteroid and alcohol-induced ONFH.

## Background

Idiopathic osteonecrosis of the femoral head (ONFH) describes atraumatic, aseptic, ischemic necrosis of the femoral head. In Japan, the incidence of morbidity due to ONFH is 17 patients per 100,000 persons per year [1]. The peak age at onset has been reported to be in the 40s and 30s for men and women, respectively, indicating a potentially significant impact on activities of daily living (ADL) since this segment of the population is expected to be at peak performance [1]. Total hip arthroplasty represents a major treatment option for ONFH in the elderly. However, joint-preserving treatments such as core decompression, vascularized bone grafting, femoral varus osteotomy, and femoral rotational osteotomy are preferred for young patients.

While conservative therapies such as non-weight bearing and muscle strengthening are available, these are not effective in most patients with ONFH. Surgical treatment options for ONFH include core decompression, transtrochanteric rotational osteotomy, and other procedures. Core decompression is less invasive, but the long-term outcome is unsatisfactory in the majority of patients [2, 3]. Rotational osteotomy is associated with good outcomes in some but not all patients, and moreover represents a relatively invasive and difficult surgical procedure [4].

Bone marrow is a source of osteoprogenitor cells, which are key elements in bone formation and fracture healing [5]. Indeed, failed or delayed fracture healing and ONFH have been reported to be repaired with autologous bone marrow transplantation. Hernigou et al. first reported the results of a prospective study on the benefits of autologous bone marrow transplantation [6, 7]. Moreover, Gangji et al. first reported the results of a controlled, double-blind trial on the efficacy of autologous bone marrow transplantation [6]. In both studies, a cell separator was used to sort and concentrate only bone marrow nucleated cells for transplantation, and the authors concluded that concentrated bone marrow transplantation improved femoral head preservation in the early stages of ONFH.

In our institution, we started concentrated autologous bone marrow aspirate transplantation (CABMAT) in 2003. The bone marrow is aspirated from the iliac crests, concentrated using a conventional manual blood bag centrifugation technique to extract buffy coats, and then injected into hips via drilling [7]. This procedure concentrates nucleated cells and platelets from clinical bone marrow aspirates (BMAs) to obtain osteogenic progenitor cells and growth factors for percutaneous transplantation to the necrotic area. We previously reported on the efficacy of CABMAT in ONFH [8, 9].

Because ONFH is a rare disease and clinical status varies significantly with stage, type, and etiology, establishing a control group is difficult. Hence, performing a comparative clinical research study represents a challenging task.

Corticosteroid-induced or alcohol-associated ONFH often affects both femoral heads. In some patients with bilateral ONFH, one of the hips is symptomatic while the other may remain asymptomatic. To our knowledge, there are limited reports regarding the management of the asymptomatic side.

The purpose of the present study was to evaluate the therapeutic effects of CABMAT for the treatment of the asymptomatic hip in patients with bilateral ONFH, relative to the natural course of asymptomatic ONFH.

## Methods

### Patients

A total of 222 patients (341 hips) with ONFH were treated with CABMAT between April 2003 and March 2013. Based on magnetic resonance imaging, bilateral and unilateral asymptomatic ONFH was diagnosed in 119 patients (238 of 341 treated hips; 69.8%) and 38 patients (38 of 341 treated hips, 11.1%), respectively. In this series, we retrospectively examined 31 of 38 patients with unilateral asymptomatic ONFH who had been followed-up for two years or more (two-year follow-up rate, 81.5%).

Among the 31 patients with unilateral asymptomatic ONFH included in the study (19 males and 12 females), the mean age was 40.0 years (range, 26–70 years). The study included 13 right hips and 18 left hips.

The study endpoint was set as the time point of the most recent follow-up or the time when the patients required additional surgery (total hip arthroplasty).

Osteonecrosis of the hip was induced by corticosteroids and alcohol in 28 and 3 hips, respectively. Corticosteroids were used in 13 of the 28 hips to manage systemic lupus erythematosus, whereas the remaining 15 patients received corticosteroids for various reasons (rheumatoid arthritis, 1; nephrotic syndrome, 1; facial nerve palsy, 1; dermatomyositis, 1; collagen disease, 1; idiopathic thrombocytopenic purpura, 2; ulcerative colitis, 1; IgA nephropathy, 2; iritis, 1; brain infarction, 1; myasthenia gravis, 1; interstitial pneumonia, 1; and primary biliary liver cirrhosis, 1).

### ONFH diagnosis

The diagnosis, classification, and staging of ONFH were based on the 2001 Japanese Orthopaedic Association (JOA) classification, which refers to findings on anteroposterior and lateral plain radiographs or magnetic resonance imaging scans [10]. Based on these criteria, ONFH was classified into four types by the location of necrotic lesions on T1-weighted or X-ray images. Type A lesions were defined as occupying the medial one third or less of the weight-bearing portion. Type B lesions were defined as occupying the medial two thirds or less of the weight-bearing portion. Type C1 lesions were defined as occupying more than the medial two thirds of the weight-bearing portion without extending laterally to the acetabular edge. Finally, type C2 lesions were defined as occupying more than the medial two thirds of the weight-bearing portion and extending laterally to the acetabular edge. Staging was based on the anteroposterior and lateral views of the femoral head on radiographs. Stage 1 was defined in hips with no specific osteonecrosis findings on radiograph but with specific findings on magnetic resonance imaging, bone scintigram, or histological examination. Stage 2 was defined in hips with demarcating sclerosis without collapse of the femoral head. Stage 3 was defined in hips with collapse of the femoral head, including crescent sign, but without joint space narrowing. Stage 3 was further divided into substages 3A and 3B, according to the extent of femoral head collapse (<3 mm and ≥3 mm, respectively). Finally, stage 4 was defined in hips with osteoarthritic changes. Preoperatively, 5, 6, 10, and 10 of 31 hips analyzed had osteonecrosis of type A, B, C1, and C2, respectively. Because all hips were asymptomatic, osteonecrosis of stage 3 or 4 was not observed. Specifically, of the 31 hips included in the study, 11 and 20 hips had ONFH stage 1 and 2, respectively.

### Bone marrow aspiration, concentration, and transplantation

Bone marrow aspiration, concentration, and transplantation were performed based on the method developed and previously performed by Sakai et al. and Yoshioka et al. [7, 8]. Specifically, the bone marrow was aspirated from both anterior iliac crests by using a bone marrow harvest needle, and subsequently transferred from the bone marrow collection kit into a collection bag. The blood fraction containing the BMA was processed by a two-step centrifugation method (KUBOTA 9800, Kubota, Japan) at room temperature. After inverse centrifugation of the blood bag at 1200 *g* for 10 min, the erythrocytes were transferred into the satellite bag until the interface between the plasma and erythrocyte layer was 15 mm from the bottom of the bag. Subsequently, using high-speed centrifugation at 3870 *g* for 7 min, plasma was transferred slowly into a satellite bag until approximately 4 cm of plasma were left on top of the buffy coat layer. Before transplantation, we typically created three multi-directional holes—central, anteromedial, and posterolateral—by percutaneous drilling with a Kirschner wire (diameter, 2.4 mm) to perforate the interface between the necrotic lesion and healthy bone. Next, core decompression was performed via the percutaneous technique with a 4.8-mm diameter trephine (Iso Medical Systems, Tokyo, Japan), which was inserted into the center of the necrotic site through the greater trochanter. Its position in the femoral head and necrotic site was monitored with biplane fluoroscopy, and transplantation was performed. After the operation, weight bearing was limited for 6 weeks, while non-weight bearing exercise was allowed.

### Clinical evaluation

Immediately before the procedure and at each follow-up, patients were evaluated in terms of the JOA hip score [11], which is an objective index of hip joint function quantified using items in three categories including (i) pain, (ii) hip range of motion, (iii) walking, and (iv) ADL performance. Clinical outcomes were defined in terms of the change in JOA hip score between the preoperative evaluation and the most recent follow-up evaluation. The JOA hip score is used as part of the preoperative and postoperative clinical assessment of hip function [12]. The 100-point scale includes subcategories for pain (40 points), range of motion (20 points), walking ability (gait; 20 points), and ADL performance (20 points), with higher scores indicating better hip function. The rate of conversion to total hip arthroplasty was also evaluated.

### Radiographic evaluation

**Fig. 1:**
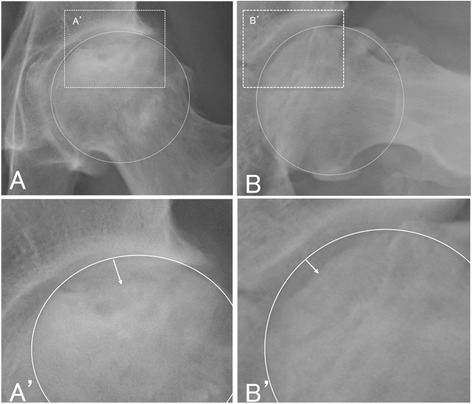
Measurement of femoral head collapse. Collapse extent is defined as the distance between the overlay circle and the collapsed femoral head (white arrow). **a** anteroposterior view. **b** lateral view (Sugioka view). **a**’ enlarged view of **a b**’ enlarged view of **b**

The anteroposterior and lateral radiographs of the affected hip were obtained at each clinical evaluation. Radiographic progression of femoral head collapse (from pre-surgery to the most recent follow-up) was evaluated in consideration of ONFH classification and staging. The extent of femoral head collapse was measured by using a template overlay of circles with increasing diameter (1-mm increments) [13] (Fig. 1). All radiographs were independently evaluated by two observers who were specialists in orthopedic surgery and authorized by the JOA.

### Statistical analysis

The JOA scores before CABMAT and at the most recent follow-up evaluation after CABMAT were compared using the paired Student’s t test. Radiological outcomes of CABMAT-treated hips were compared against those of untreated hips (natural course of ONFH) and against those of hips treated with core decompression, and such comparisons used χ^2^ tests, Fisher’s tests, and log rank test (for survival curves). Data were presented as mean ± standard deviation. A *P*-value of <0.05 was considered to indicate statistical significance. All statistical analyses were performed with IBM SPSS Statistics version 19.0 (IBM Corporation, Armonk, USA).

## Results

### Mean follow-up duration

Overall, the mean follow-up period was 5.8 years (range, 2.0–6.9 years). On stratification, according to type of osteonecrosis, the mean and range of follow-up duration were 4.1 (2.0–6.5), 3.0 (2.0–3.5), 4.2 (2.0–6.4), and 5.8 (3.7–6.9) years for ONFH of type A, B, C1, and C2, respectively.

### JOA score

The mean and range of the JOA hip score was 88 (72–100) and 92 (45–100) points before surgery and at the most recent follow-up evaluation, respectively. One patient who underwent total hip arthroplasty of the contralateral side was excluded from this evaluation. While the mean JOA score improved following CABMAT, the improvement was not statistically significant (*P ≥* 0.05, Student’s t test). The pain score was 40 (40–40) and 36 (20–40) points before surgery and at the most recent follow-up, respectively. Although the pain score decreased significantly after surgery (*P* < 0.05), only 6% (2/31) of patients reported pain during walking, whereas the other patients reported feeling no pain or slight discomfort in the affected hip.

**Fig. 2:**
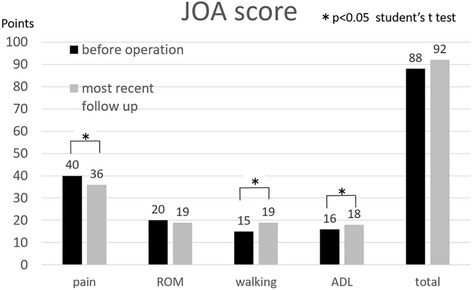
Assessment of hip function in terms of the Japanese Orthopaedic Association (JOA) hip score. The JOA hip score is measured on a 100-point scale comprised of categories for pain (40 points), range of movement (ROM; 20 points), walking ability (gait; 20 points), and activities of daily living (ADL; 20 points), with higher score indicating better hip function. JOA score of walking and ADL points improved significantly at the most recent follow-up examination

The JOA walking and ADL scores, respectively, were 15 (10–20) and 19 (10–20) points before surgery, but 16 (10–20) and 18 (6–20) points after surgery, indicating significant improvement (*P* < 0.05, Student’s t test). Finally, the JOA range of motion score was 20 (17–20) and 19 (9–20) points before and after surgery, respectively, which did not indicate a significant change following surgery (Fig. 2).

### Rate of conversion to total hip arthroplasty

A total of 9.6% (3/31) of patients underwent total hip arthroplasty at an average of 33 months after surgery.

### Radiographic progression of femoral head collapse after CABMAT

Among hips with ONFH of types C1 and C2, 6 of 10 hips (60%) and 5 of 10 hips (50%), respectively, exhibited progression of femoral head collapse. The extent of collapse is listed in Table 1.

**Table 1:** Relationship between hip lesion type and progression of femoral head collapse. Collapse rate and extent were higher for osteonecrosis of type C than for osteonecrosis of types A or B

Collapse distance (mm)	Type				Total
	A	B	C1	C2	
0	5	4	4	5	18(58%)
0–3	0	2	2	3	7(23%)
3–5	0	0	3	1	4(13%)
5≦	0	0	1	1	2(6%)
Collapsed hip	0/5	2/6	6/10	5/10	13/31
Collapse rate (%)	0%	33%	60%	50%	(42%)

## Discussion

### CABMAT vs natural course

By comparing the outcomes of CABMAT treatment for asymptomatic, pre-collapsed ONFH with those associated with the natural course of ONFH, we could verify that CABMAT changed the natural course of the disease. Compared to the natural course and the outcomes of core decompression reported in the past, the outcomes of ONFH treated with CABMAT were better, as it is discussed in detail in the following paragraphs. Though CABMAT was not 100% successful, we found clear evidence that such a procedure can alter the natural course of the disease in some patients with ONFH.

We further discuss our findings in the context of current knowledge in the field of ONFH management. Nevertheless, since it is not possible to accurately match our study sample to those from previous investigations in terms of extent and location of ONFH, the comparisons should be considered qualitative.

#### CABMAT vs natural course according to ONFH type

**Fig. 3:**
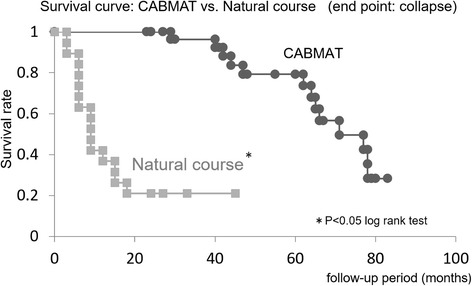
Survival free from femoral head collapse in ONFH: CABMAT vs natural course. Curves describe outcomes after concentrated autologous bone marrow aspirate transplantation (CABMAT; present study) and outcomes of the natural course of the disease (taken from reference [14]). ONFH, osteonecrosis of the femoral head

In our study, the overall collapse rate (i.e., for all types of ONFH) was 42%, noted on a mean follow-up of 5.8 years. The rates of femoral head collapse noted in hips with ONFH of type A, B, C1, and C2 were 0 of 5 hips (0%), 2 of 6 hips (33%), 6 of 10 hips (60%), and 5 of 10 hips (50%), respectively. Koo and Kim reported a collapse rate of 79% (15/19) in the untreated hip, recorded on a follow-up of at least 2 years [14]. While the collapse rate noted in our study was significantly better than that reported by Koo and Kim (log rank test, *P* < 0.01) (Fig. 3), it is worth noting that Koo and Kim used a different definition of the necrotic area. However, since 21% (4/19) of hips evaluated by Koo and Kim were symptomatic, a simple comparison against the outcomes of our study is difficult.

#### CABMAT vs natural course in ONFH of types C1 or C2

**Fig. 4:**
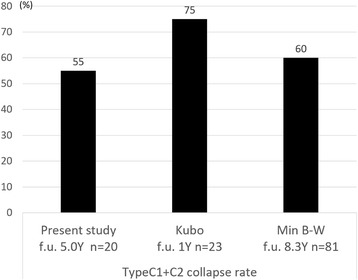
Comparison of femoral head collapse rates in ONFH of types C1 and C2. Data describes outcomes after concentrated autologous bone marrow aspirate transplantation (CABMAT; present study) and outcomes of the natural course of the disease (taken from references [14, 15]). ONFH, osteonecrosis of the femoral head

In our study, the collapse rate in hips with ONFH of types C1 or C2 was 55%, recorded on a mean follow-up of 5.0 years. Several studies have focused on the natural course of ONFH of type C, with collapse rates ranging from 60% to 76% in asymptomatic hips; such studies had a mean follow-up of 1 year [15], and 8.3 years [16]. The mean collapse rate noted in our present study (55%) was relatively lower than previously reported rates [15, 16] (Fig. 4).

#### CABMAT vs natural course in ONFH of type C2

In our study, the collapse rate in hips with ONFH of type C2 was 50%, noted on a mean follow-up of 5.0 years. Min et al. reported a collapse rate of 86% in asymptomatic, untreated femoral heads, on a mean follow-up of 8.3 years [16]. The collapse rate reported in our study for CABMAT-treated hips was significantly better than that reported by Min et al. in untreated femoral heads (Fisher’s exact test, *P* < 0.05).

#### CABMAT vs core decompression

In our study, the femoral head collapse rate in hips with ONFH of types A–C2 was 42%, recorded on a mean follow-up of 5.0 years. Based on several studies that reported on the outcome of core decompression for ONFH, the collapse rate ranged from 43% to 82% [2, 3, 14, 17–19].

**Fig. 5:**
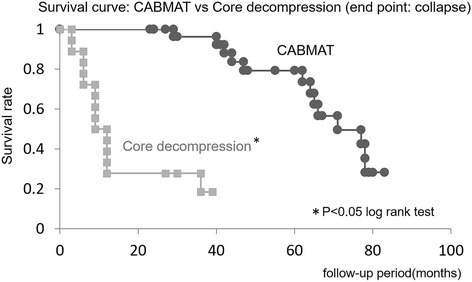
Survival free from femoral head collapse in ONFH: CABMAT vs core decompression. Curves describe outcomes after concentrated autologous bone marrow aspirate transplantation (CABMAT) and outcomes after core decompression. ONFH, osteonecrosis of the femoral head

The collapse rate noted in our study was significantly better than the rates reported by Koo and Kim (log rank test, *P* < 0.05) (Fig. 5) [14] or by Learmonth et al. (Fisher exact test, *P* < 0.05) [17]. Additionally,

Mont et al. [18] and Song et al. [19] reported clinical success rates of 71% (34/45 hips) and 66% (108/163 hips), respectively, after core decompression. On defining clinical success as a JOA hip score ≥ 80 points and no requirement for further surgery, which is the definition used in these previous studies, the success rate in the present study is 81% (25/31 hips), which is only marginally better than the success rates reported by Mont et al. and Song et al.

#### CABMAT vs other procedures involving bone marrow transplantation

Based on several studies that reported on the conversion rate to total hip arthroplasty after treatment with bone marrow transplantation, the conversion rate is expected to range from 6.2% to 15.4% [6, 8, 12] which covers the value noted in our study (9.6%, 3/31). Specifically, Hernigou et al. reported a conversion rate of 6.2% (9/189) for asymptomatic hips (ONFH stage 1 or 2) over a mean follow-up period of 7 years after transplantation of bone marrow processed using a cell separator and centrifuge for concentration [6]. Gangji et al. reported a conversion rate of 15.4% (2/13) for asymptomatic hips (ONFH stage 1 or 2) over a mean follow-up period of 5 years after transplantation of bone marrow processed using a cell separator and centrifuge for concentration [8]. In our study, 9.6% (3/31) of the patients underwent total hip arthroplasty at an average of 33 months after surgery, and this conversion rate was not significantly different from those reported in the studies by Hernigou et al. and Gangji et al. (*P* > 0.05, χ^2^ test).

#### Mechanism of effectiveness of CABMAT

The exact mechanism of ONFH induced by corticosteroids or alcohol remains unclear. Hernigou et al. suggested that ONFH may be a disease of bone cells and mesenchymal stem cells [20], since the numbers and activity of these cells have been shown to be decreased in both the hematopoietic and stromal compartments of the bone marrow in patients with ONFH [6, 7]. In addition, the number of progenitor cells was significantly lower in patients undergoing corticosteroid therapy than in patients who had a different underlying etiology [21].

One of the mechanisms underlying CABMAT effectiveness is presumed to be transplantation of bone marrow graft, which involves introduction of new stem/progenitor cells to an area with deficient or dead stem/progenitor cells (necrotic lesion), accelerating bone formation. On the other hand, it is believed that the mechanism underlying the therapeutic effect of CABMAT with multiple drillings involves core decompression [22]. Specifically, core decompression decreases the intraosseous pressure and opens a channel into the necrotic area, which is speculated to alter the natural course of necrosis and encourage revascularization and repair. We drilled three channels to induce migration of nucleated cells and platelets to the necrotic site for bone regeneration [23].

While CABMAT is a less-invasive surgical option compared with total hip arthroplasty and osteotomy, it is more invasive than core decompression, since it requires aspiration from the iliac crest. Nevertheless, no infection or tumorigenic transformation was noted in our patients treated with CABMAT.

### Limitations

This study has several limitations. First, because this was a retrospective study and because ONFH is a rare disease with large variations in clinical status (stage, type, etiology), defining a control group was difficult. The lack of a control group represents a limitation of this study. Furthermore, we used a small sample size (31 patients). To validate the efficacy of CABMAT, prospective studies are warranted, preferably in the form of multicenter, randomized clinical trials comparing CABMAT outcomes with the natural course of ONFH and with the outcomes of core decompression. Additional study limitations include the lack of adequate randomization and matching based on ONFH stage, ONFH type, sex, and age, which should be considered in future investigations.

## Conclusions

In our study, the collapse rate after CABMAT was lower than the rate previously reported to be associated with the natural course of asymptomatic ONFH. These findings suggest that early diagnosis of ONFH (i.e., before femoral head collapse) and early intervention with CABMAT could improve the clinical outcomes of ONFH. Because CABMAT is a low-invasive therapy with relatively good clinical outcomes, it would be the first-choice treatment for early-stage ONFH.

## Abbreviations

ADL: Activities of daily living
BMA: Bone marrow aspirate
CABMAT: Concentrated autologous bone marrow aspirate transplantation
JOA: Japanese Orthopaedic Association
ONFH: Osteonecrosis of femoral head

## References

[1] Fukushima W, Fujioka M, Kubo T, Tamakoshi A, Nagai M, Hirota Y (2010). Nationwide epidemiologic survey of idiopathic osteonecrosis of the femoral head. Clin Orthop Relat Res.

[2] Stulberg BN, Bauter TW, Belhobek G (1990). Making core decompression work. Clin orthop Relat. Res.

[3] Kristensen KD, Pedersen NW, Kiæer T, Starklint H (1991). Core decompression in femoral head osteonecrosis. 18 stage I hips followed up for 5 years. Acta Orthop Scand.

[4] Sugioka Y, Hotokebuchi T, Tsutsui H (1992). Transtrochanteric anterior rotational osteotomy for idiopathic and steroid-induced necrosis of the femoral head: indications and long-term results. Clin Orthop Relat Res.

[5] Connolly J, Shinell R (1986). Percutaneous marrow injection for an ununited tibia. Nebr Med J.

[6] Gangji V, Hauzeur J-P, Matos C (2004). Treatment of osteonecrosis of the femoral head with implantation of autologous bone-marrow cells. J Bone Joint Surg Am.

[7] Sakai S, Mishima H, Ishii T, Akaogi H, Yoshioka T, Uemura T (2008). Concentration of bone marrow aspirate for osteogenic repair using simple centrifugal methods. Acta Orthop.

[8] Yoshioka T, Mishima H, Akaogi H, Sakai S, Li M, Ochiai N (2011). Concentrated autologous bone marrow aspirate transplantation treatment for corticosteroid-induced osteonecrosis of the femoral head in systemic lupus erythematosus. Int Orthop.

[9] Hyodo K, Yoshioka T, Sugaya H, Akaogi H, Aoto K, Wada H (2017). Predicting risk factors of total hip arthroplasty conversion after concentrated autologous bone marrow aspirate transplantation for the treatment of idiopathic osteonecrosis of the femoral head: a retrospective review of 213 hips at a mean follow-up of 5 years. J Hip Surg.

[10] Sugano N, Atsumi T, Ohzono K (2002). The 2001 revised criteria for diagnosis, classification, and staging of idiopathic osteonecrosis of the femoral head. J Orthop Sci.

[11] Ogawa R, Imura S (1995). Evaluation chart of hip joint functions. Nippon Seikeigeka Gakkai Zasshi.

[12] Nishino T, Mishima H, Kawamura H, Shimizu Y, Miyakawa S, Ochiai N (2013). Follow-up results of 10-12 years after total hip arthroplasty using cementless tapered stem — frequency of severe stress shielding with synergy stem in japanese patients. J Arthroplast.

[13] Aaron RK, Lennox D, Bunce GE (1989). Conservative treatment of osteonecrosis of the femoral head. Comparison of core decompression and pulsing electromagnetic fields. Clin Orthop.

[14] Koo K, Kim R (1996). Preventing collapse in early osteonecrosis of the femoral head. A randomised clinical trial of core decompression. J Bone Joint Surg Br..

[15] Kubo T, Yamazoe S, Sugano N, Fujioka M, Naruse S, Yoshimura N (1997). Initial MRI findings of non-traumatic osteonecrosis of the femoral head in renal allograft recipients. Magn Reson Imaging.

[16] Min BW, Song KS, Cho CH, Lee SM, Lee KJ (2008). Untreated asymptomatic hips in patients with osteonecrosis of the femoral head. Clin Orthop Relat Res.

[17] Learmonth ID, Maloon S, Dall G (1990). Core decompression for early atraumatic osteonecrosis of femoral head. J Bone Joint Surg Br..

[18] Mont MA, Ragland PS, Etienne G (2004). Core decompression of the femoral head for osteonecrosis using percutaneous multiple small-diameter drilling. Clin Orthop Relat Res.

[19] Song WS, Yoo JJ, Kim Y-M, Kim HJ (2007). Results of multiple drilling compared with those of conventional methods of core decompression. Clin Orthop Relat Res.

[20] Hernigou P, Poignard A, Manicom O, Mathieu G, Rouard H (2005). The use of percutaneous autologous bone marrow transplantation in nonunion and avascular necrosis of bone. J Bone Joint Surg Br.

[21] Gao Y, Zhang C (2010). Cytotherapy of osteonecrosis of the femoral head: a mini review. Int Orthop.

[22] Mont M, Fairbank A, Petri M (1997). Core decompression for osteonecrosis of the femoral head in systemic lupus erythematosus. Clin Orthop Relat Res.

[23] Lee H, Huang G, Chiang H (2003). Multipotential mesenchymal stem cells from femoral bone marrow near the site of osteonecrosis. Stem Cells.

